# Sox10 Activity and the Timing of Schwann Cell Differentiation Are Controlled by a Tle4-Dependent Negative Feedback Loop

**DOI:** 10.3390/ijms25105234

**Published:** 2024-05-11

**Authors:** Tim Aberle, Anna Walter, Sandra Piefke, Simone Hillgärtner, Hannah M. Wüst, Michael Wegner, Melanie Küspert

**Affiliations:** Institut für Biochemie, Friedrich-Alexander-Universität Erlangen-Nürnberg, Fahrstrasse 17, 91054 Erlangen, Germany; tim.aberle@fau.de (T.A.);

**Keywords:** Sox10, HMG, glia, transcriptional regulation, Schwann cells

## Abstract

The HMG-domain containing transcription factor Sox10 plays a crucial role in regulating Schwann cell survival and differentiation and is expressed throughout the entire Schwann cell lineage. While its importance in peripheral myelination is well established, little is known about its role in the early stages of Schwann cell development. In a search for direct target genes of Sox10 in Schwann cell precursors, the transcriptional co-repressor Tle4 was identified. At least two regions upstream of the *Tle4* gene appear involved in mediating the Sox10-dependent activation. Once induced, Tle4 works in tandem with the bHLH transcriptional repressor Hes1 and exerts a dual inhibitory effect on Sox10 by preventing the Sox10 protein from transcriptionally activating maturation genes and by suppressing Sox10 expression through known enhancers of the gene. This mechanism establishes a regulatory barrier that prevents premature activation of factors involved in differentiation and myelin formation by Sox10 in immature Schwann cells. The identification of Tle4 as a critical downstream target of Sox10 sheds light on the gene regulatory network in the early phases of Schwann cell development. It unravels an elaborate regulatory circuitry that fine-tunes the timing and extent of Schwann cell differentiation and myelin gene expression.

## 1. Introduction

Schwann cells, the myelinating glial cells of the peripheral nervous system (PNS), play important roles in supporting neuronal axons with guidance and trophic cues and by insulating them with multilayered myelin sheaths allowing the fast saltatory conduction of action potentials. In contrast to their central nervous system (CNS) equivalent, the myelinating oligodendrocytes, Schwann cells are very plastic and able to de- and redifferentiate efficiently after injury. A complex and unique network of pro-and anti-differentiative regulatory factors is the prerequisite for the correct timing of developmental PNS myelination as well as for the regeneration of myelinated PNS axons. A second major difference between oligodendrocytes and Schwann cells lies in different dependencies on the HMG-box transcription factor Sox10. Sox10 is expressed throughout the whole Schwann cell and oligodendrocyte lineage (for further information see [1]). Whereas oligodendrocyte precursor cells display surprisingly normal developmental features in the absence of Sox10 and only show differentiation defects, Schwann cells rely on Sox10 functions throughout their development. In late phases of Schwann cell maturation and PNS myelination Sox10 was shown to be a key activator of important transcriptional regulators of Schwann cell differentiation, such as Oct6 and Egr2 [2,3,4,5]. Together with them, Sox10 synergistically activates the expression of structural myelin genes, such as *Mpz*, and genes of cell adhesion and gap junction molecules, such as *Nfsc*, *Nrcam*, and *Cx32* ([6]; for further information, see [1]). Neither myelin proteins nor myelin sheaths are detectable in the murine PNS after the deletion of Sox10 in immature Schwann cells (for further information, see [1]). Additionally, peripheral demyelinating neuropathy is associated with mutations of Sox10 in human PCWH (peripheral demyelinating neuropathy, central dysmyelinating leukodystrophy, Waardenburg syndrome, and Hirschsprung disease) patients ([7,8,9,10,11,12,13]; for further information, see [1]). This reflects the essential character of Sox10 during Schwann cell differentiation and PNS myelination. Apart from late functions, Sox10 was also shown to be essential for Schwann cell identity and survival, since constitutive deletion of Sox10 in mice leads to the complete loss of Schwann cell precursors from the PNS and later conditional deletion leads to the ectopic expression of non-glial markers in Sox10-deleted immature Schwann cells (for further information, see [1]). Despite the obvious functions of Sox10 in the early phases of Schwann cell development, not much is known about the relevant early targets. Additionally, there are conflicting findings on this topic, since Sox10 was shown to bind to and activate gene regulatory regions of inhibitors of peripheral myelination, while other publications show repression of exactly the same genes by Sox10 overexpression in concert with the Hippo pathway downstream effector Taz [14,15,16]. To further clarify this discrepancy and to identify previously unknown target genes of Sox10 that are specifically enriched in the early phases of Schwann cell development, we analyzed publicly available data sets. Selection criteria were (I) a high expression in undifferentiated primary rat Schwann cells with a decreased expression upon in vitro differentiation [17], (II) genomic Sox10 occupancy in open chromatin regions identified by ChIP-sequencing for the H3K27ac active histone mark using early postnatal rat sciatic nerve tissue [18], and (III) published or predicted functions as transcriptional regulators.

As a result of these studies, we focused on the transcriptional co-repressor Tle4/Grg4 as a potential Sox10 target with high expression in proliferating Schwann cell precursors and immature Schwann cells and low expression in mature myelinating Schwann cells in vitro and in vivo. Tle4 was initially identified as a mammalian orthologue of the *Drosophila melanogaster* corepressor Groucho and is itself devoid of a DNA-binding domain but recruited by DNA-binding transcription factors to its target genes for the repression of productive transcription. Tle proteins fulfil this function through different modes of action, depending on their interacting transcription factor and the genomic context, and are important regulators of mammalian development and tumorigenesis in a variety of cell types [19,20]. Here, we aimed to analyze the mutual effector–target relationship between Sox10 and Tle4 and its functional relevance in the early phases of Schwann cell development to shed light on the complex regulatory interactions leading to the maintenance of the immature Schwann cell state and the correct timing of Schwann cell differentiation. We tackled those questions using a combination of in vivo and in vitro expression studies, reporter gene assays, and Tle4 functional analyses using primary Schwann cell culture.

## 2. Results

### 2.1. Tle4 Is Predominantly Expressed in Schwann Cell Progenitors and Early Immature Schwann Cells

**Figure 1:**
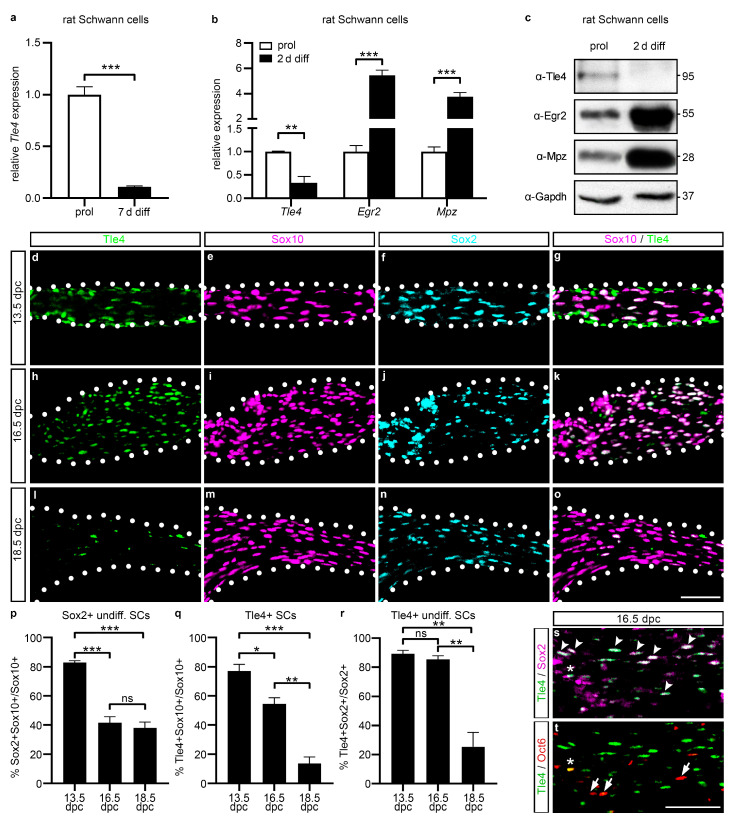
Stage-specific expression of Tle4 in undifferentiated Schwann cells. (**a**) Relative *Tle4* transcript expression in primary rat Schwann cell cultures in proliferative conditions and after cAMP treatment to induce terminal differentiation for 7 days (7 d diff, modified from [17]). (**b**,**c**) Relative expression levels of *Tle4*, *Egr2*, and *Mpz* transcripts (**b**) or proteins (**c**) in primary rat Schwann cell cultures in proliferative conditions (prol) or after two days of in vitro differentiation (2 d diff). (**b**) Transcript levels were normalized to *Gapdh* and *Rpl8* levels and the proliferative conditions were arbitrarily set to 1. Data represent mean values ± SEM. Statistical analyses were performed with Student’s two-tailed *t*-tests (** *p* ≤ 0.01, *** *p* ≤ 0.001). (**c**) Western Blot was performed on whole cell extracts of primary rat Schwann cells with antibodies or antisera directed against Tle4, Egr2, and Gapdh (Gapdh blots refer to Tle4 or Egr2 blots depicted above). Numbers on the right indicate the detected molecular weight in kDa. (**d**–**o**,**s**,**t**) Representative immunohistochemical stainings of transverse spinal nerve sections on forelimb level from wildtype mice at 13.5 days *postcoitum* (dpc), 16.5 dpc, and 18.5 dpc using antibodies or antisera directed against Tle4 (green), Sox10 (magenta), Sox2 (cyan (**f**,**j**,**n**); magenta in (**s**)), and Oct6 (red). Arrowheads mark Sox2/Tle4-positive, Oct6-negative cells, asterisks mark Sox2/Oct6/Tle4-positive cells, and arrows mark Oct6-positive, Tle4-negative cells (**s**,**t**). Scale bar: 50 µm. (**p**–**r**) Quantifications of immunohistochemical stainings represented in (**d**–**o**). Percentages of Sox2/Sox10-positive undifferentiated (undiff.) Schwann cells (SCs) of all Sox10-positive SCs (**p**), Tle4-positive cells among all Sox10-positive SCs (**q**) or all Sox2-positive undiff. SCs (**r**) are depicted as mean ± SEM. Statistical analyses were performed using Student’s two-tailed *t*-tests (ns *p* > 0.05, * *p* ≤ 0.05, ** *p* ≤ 0.01, *** *p* ≤ 0.001).

RNA-sequencing data by Camarena and colleagues [17] argue that *Tle4* transcripts are highly enriched in Schwann cell precursors and immature Schwann cells, subsequently referred to as undifferentiated Schwann cells, and strongly decreased in mature myelinating Schwann cells after 7 days of in vitro differentiation (7 d diff) (Figure 1a). To confirm these data, we performed qRT PCR for *Tle4* on cultured primary rat Schwann cells in proliferative conditions and after two days of in vitro differentiation. Levels of *Tle4* transcripts were substantially reduced after 2 days of in vitro differentiation, whereas markers of terminal Schwann cell differentiation, such as *Egr2* and *Mpz*, were increased on the transcript level (Figure 1b). Western Blot analysis on whole cell extracts of primary rat Schwann cells cultured in the same conditions showed similar antagonistic regulation of the differentiation marker Egr2 and Tle4 on the protein level (Figure 1c). Additionally, we analyzed the occurrence of the Tle4 protein in spinal nerves of wildtype mice at different prenatal stages. Tle4 expression was detectable in the majority of Sox10-positive cells of the Schwann cell lineage at 13.5 days *post coitum* (dpc), when Schwann cells were still in the Oct6-negative but Sox2-positive Schwann cell precursor stage (Figure 1d–g,p–r). However, expression faded to 54% of all Sox10-positive Schwann cells at 16.5 dpc, when Sox2/Oct6-positive immature Schwann cells and Sox2-negative, Oct6-positive pro-myelinating Schwann cells emerged (Figure 1h–k,p–t). Tle4 was merely present in 14% of Sox10-positive Schwann cells at 18.5 dpc, when the first Schwann cells reach the Egr2-positive myelinating Schwann cell stage (Figure 1l–r, [21]). The overlapping expression of the Tle4 protein with Sox2 and only marginal overlap with Oct6 supports the assumption deduced from the qRT PCR and Western Blot analyses on primary rat Schwann cells (Figure 1b,c) that Tle4 is solely expressed in the Schwann cell precursors and early immature Schwann cells.

### 2.2. Tle4 Expression Is Regulated by Sox10 in Undifferentiated Schwann Cells

**Figure 2:**
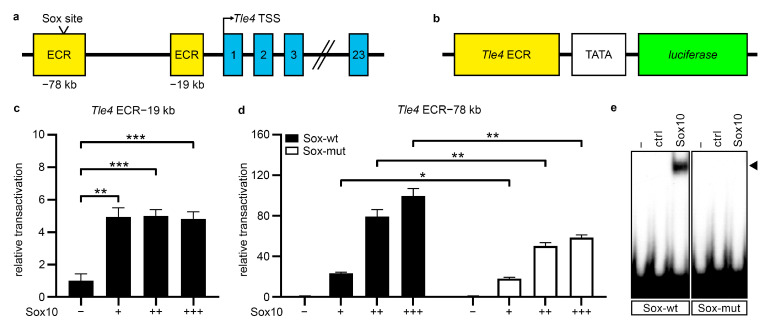
Sox10 directly binds to and activates two newly identified *Tle4* upstream enhancers. (**a**) Localization of the evolutionary conserved regions (ECRs, yellow boxes) and the conserved Sox binding site upstream of the mouse *Tle4* gene (exons depicted as blue boxes). (**b**) Schematic representation of the luciferase reporter constructs used in (**c**,**d**). The *Tle4* ECRs (yellow box) and a minimal promoter (TATA, white box) control the expression of a firefly *luciferase* gene (green box). (**c**,**d**) Transient transfections of the *Tle4* ECR luciferase reporter constructs were performed in Sox10-negative Neuro2a neuroblastoma cells. The reporters were co-transfected with either a control plasmid (−) or increasing amounts of Sox10 (+, ++, +++). Reporter gene expression for the *Tle4* ECR-19 kb (**c**) and the wildtype (Sox-wt) or mutated (Sox-mut) *Tle4* ECR-78 kb constructs (**d**) was measured, and effector-dependent activation rates are presented as relative transactivation ± SEM with relative light units of control plasmid-transfected cells arbitrarily set to 1. Statistical analyses were performed using Student’s two-tailed t-tests (* *p* ≤ 0.05, ** *p* ≤ 0.01, *** *p* ≤ 0.001). (**e**) EMSAs were performed with radiolabeled probes for the wildtype Sox consensus site (Sox-wt) in ECR-78kb and a mutated (Sox-mut) version without cell extracts (−) or cell lysates from HEK293T cells transfected with empty pCMV5 vector (ctrl) or pCMV5-Sox10-MIC (Sox10) vector. Specific bands for Sox10 are marked by an arrowhead.

Based on published ChIP-sequencing data sets [18] Sox10 binds to genomic regions in close vicinity to the *Tle4* gene in rat sciatic nerve tissue at postnatal day (P)14. A screening for evolutionary conservation (Dcode ECR browser; https://ecrbrowser.dcode.org/, accessed on 16 October 2019) was performed. Using a minimum length of 300 bp and a base identity of at least 75% between human, mouse, and rat as the selection criteria, we identified two evolutionary conserved regions (ECRs) and potential gene regulatory regions of *Tle4*. These were located at −19 kb and −78 kb relative to the *Tle4* transcriptional start site in the rat genome (Rn6, Figure 2a) and showed enrichment of Sox10 binding in ChIP-sequencing experiments [18]. The fragments used for further analysis in case of the ECR-19 kb encompassed 800 bp, and in case of the ECR-78 kb, 1042 bp. To analyze their activity as gene regulatory regions as well as their transcriptional regulation by Sox10, luciferase reporter gene assays were performed using constructs harboring the respective ECR in front of the *ß-globin* minimal promoter and the luciferase open reading frame (Figure 2b). Since primary Schwann cells endogenously express Sox10 and are not easily transfected, the Sox10-negative but neural crest derived Neuro2a neuroblastoma cell line was used for luciferase reporter assays. Both ECRs were significantly activated by Sox10. In contrast to ECR-78 kb, which was strongly activated by Sox10 in a dose-dependent manner (100 ± 7.5-fold at highest Sox10 amounts), ECR-19.5 kb was only mildly activated by Sox10 to a maximum of 5 ± 0.4-fold (Figure 2c,d). This indicates a much stronger relevance of ECR-78 kb for Sox10-dependent regulation of *Tle4* expression. To identify Sox10 binding sites in this enhancer, we screened for conserved Sox consensus binding motifs (5′-(A/T)(A/T)CAA(A/T)G-3′) and found one evolutionary conserved Sox10 binding site. Mutation of this site to a non-consensus motif resulted in significantly lower activation of ECR-78 kb by Sox10 in luciferase reporter assays (Figure 2d). To prove direct binding of Sox10 to the identified Sox consensus motif, electrophoretic mobility shift assays (EMSAs) were performed using a Sox10 protein encompassing only the N-terminal amino acids 1-189 including the HMG DNA binding domain (Sox10 aa1-189, Sox10-MIC) [22]. Sox10 specifically bound to a 23 bp oligonucleotide containing the putative Sox site with its flanking regions, and mutation of the Sox motif abolished Sox10 binding to this site in good agreement with the results from luciferase assays (Figure 2e). This strongly suggests a positive effector–target relationship between Sox10 and Tle4.

### 2.3. Tle4 Interacts with Hes1 to Repress Sox10-Dependent Activation of Maturity Factors and Myelin Proteins

**Figure 3:**
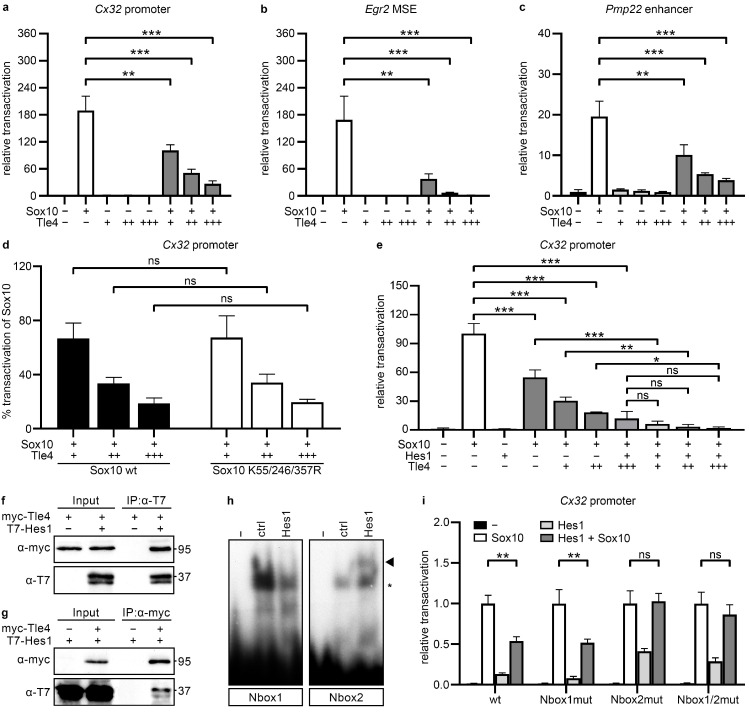
Tle4 and Hes1 bind to and repress Sox10-dependent gene regulatory regions of Schwann cell differentiation genes and peripheral myelin genes. (**a**–**e**) Luciferase assays in Neuro2a cells transiently transfected with reporter constructs containing the regulatory regions *Cx32* promoter (**a**,**d**,**e**), *Egr2 MSE* (**b**), and *Pmp22* intronic enhancer (**c**) upstream of the firefly luciferase open reading frame. Co-transfections with empty expression vectors (−) or varying amounts of expression vectors (+, ++, +++) for Sox10, Tle4, and Hes1 or a combination were performed as indicated. In (**d**) either the wildtype Sox10 (wt) or a non-sumoylatable Sox10 variant (K55/246/357R) was used. Reporter gene expression was measured and transfections with empty expression vectors were arbitrarily set to 1, except in (**d**) where Sox10 activation alone was set to 100% for a better comparison of the specific effects in Tle4 repression (**d**). Data indicate mean ± SEM and statistical analysis was performed with a one-way ANOVA with Tukey’s multiple comparisons tests (ns *p* > 0.05, * *p* ≤ 0.05, ** *p* ≤ 0.01, *** *p* ≤ 0.001). (**f**,**g**) Co-immunoprecipitations were performed with HEK293T lysates overexpressing either myc-Tle4, T7-Hes1 or a combination. Numbers on the right indicate the molecular weight in kDa. (**f**) Precipitations were performed with either the anti-T7 antibody (**f**) or anti-myc antibody (**g**). T7-Hes1 was detected with an anti-T7 antibody and myc-Tle4 was detected with an anti-myc antibody. (**h**) EMSAs were performed with radiolabeled probes containing the Nbox consensus sites Nbox1 and Nbox2 present in the analyzed *Cx32* promoter fragment without cell extracts (−) or with cell lysates from HEK293T cells transfected with the pCMV5 empty vector (ctrl) or pCMV5-T7-Hes1 vector (Hes1). Specific bands for Hes1 are marked by an arrowhead and unspecific bands by an asterisk. (**i**) Luciferase assays were performed in Neuro2a cells transiently transfected with luciferase reporter constructs containing the regulatory region *Cx32 prom* in a wildtype variant (wt) or with mutations in the Nbox1 (Nbox1mut), Nbox2 (Nbox2mut) or both Nbox consensus sites (Nbox1/2mut). Co-transfections with empty pCMV5 expression vector (−), expression vectors for Sox10 and Hes1 or a combination were performed as indicated. Reporter gene expression was measured and mean relative transactivation rates ± SEM are depicted. Transfections with Sox10 vector only were arbitrarily set to 1. Statistical analysis was performed with a two-way ANOVA with Tukey’s multiple comparisons tests between meaningful groups (ns *p* > 0.05, ** *p* ≤ 0.01).

Sox10 acts as a direct activator of a variety of Schwann cell maturation and myelin genes by direct binding and activation of their gene regulatory regions [5,23,24]. We screened a selection of these published gene regulatory regions (*Cx32* promoter, *Pmp22* intronic enhancer, and *Egr2* enhancer *MSE*) for regulatory effects of the corepressor Tle4 on Sox10 activation using luciferase reporter assays in Sox10-negative Neuro2a cells. We found a robust dose-dependent repression of the Sox10 transactivation capacity by Tle4 for all of the analyzed gene regulatory regions (Figure 3a–c). Sox10 activation of the *Egr2 MSE* enhancer was completely blocked by co-expression of Tle4 in higher doses. Sox10 activation of the *Cx32* promoter and the *Pmp22* enhancer was reduced to only 14% or 20% in high Tle4 conditions. This indicates a strong inhibitory effect of Tle4 on Sox10-dependent activation of Schwann cell differentiation genes.

To further elucidate the signaling pathways or interaction partners used by Tle4 to exert this repressive function, we tested different known Tle4 interacting proteins and pathways in luciferase reporter assays. We focused on the *Cx32* promoter construct, which showed an intermediate repressive effect by Tle4 on Sox10 activation.

In melanocytes, Tle4 was shown to interact with the Sox10-related transcription factor Sox9 and to change its function from a transcriptional activator to a transcriptional repressor of the *Mitf* gene transcription. This interaction was strictly dependent on posttranslational modification of Sox9 by sumoylation at several amino acid residues [25]. These amino acids are evolutionary conserved as lysine residues K55, K246, and K357 in Sox10 and can also be sumoylated [26]. In the case of Sox10, mutation of these residues to non-sumoylatable alanine residues has been reported to increase the transactivation capacity of Sox10 and its synergistic function with Pax3 and Egr2 on the joint target genes *Mitf* and *Cx32* [26]. In our experimental setup mutation of all three lysine residues of Sox10 to equally non-sumoylatable arginine residues did not significantly alter Sox10 activation of the *Cx32* promoter nor the repressive effect of Tle4 on Sox10 activation (Figure 3d). This indicates that sumoylation of Sox10 is not a prerequisite for repression by Tle4 in Schwann cells.

A transcriptional repressor described to interact with Tle4 in inflammatory responses of macrophages is the bHLH protein Hes1 [27]. Unlike other Hes family members, *Hes1* transcripts are highly expressed in undifferentiated primary rat Schwann cells and strongly downregulated upon the induction of Schwann cell differentiation via cAMP treatment [17]. Therefore, Hes1 was selected as the most probable Tle4 interactor in undifferentiated Schwann cells. In several cell types of the nervous system, including the Schwann cell lineage, Hes protein expression is induced by active Notch signaling in a highly conserved and oscillatory manner [28,29,30,31,32,33]. Additionally, few Notch-independent upstream regulatory mechanisms were disclosed for Hes1 within the last decades in other cell types, such as activation of the JNK pathway [34].

In contrast to activating bHLH proteins, Hes1 preferentially binds to the N-box consensus motif (5′-CACGAG-3′) [35] and interferes with transcriptional activation of its target genes. Upon co-transfection of Hes1 with Tle4 in luciferase reporter gene assays on the *Cx32* promoter, we indeed detected additive repressive effects of both proteins on the Sox10 transactivation capacity (Figure 3e). Tle4 repressed Sox10-mediated activation of this promoter in a dose-dependent manner to a minimum of 18% ± 1% and Hes1 to 12% ± 7% of the level achieved with Sox10 alone. In combination, Tle4 and Hes1 showed a stronger repressive effect than either Tle4 or Hes1 alone and reduced Sox10 activation to a minimum of 2% ± 1%. Interaction of overexpressed myc-Tle4 and T7-Hes1 proteins was shown in protein extracts from transfected HEK293T cells by co-immunoprecipitation of Hes1 with Tle4 (Figure 3f). Similarly, Tle4 was also detected in the precipitates obtained with antibodies directed against the tagged Hes1 protein (Figure 3g). These findings support the assumption that Hes1 can recruit the Tle4 corepressor to its target regions to repress the precocious Sox10-dependent induction of Cx32 and possibly other maturity factors in immature Schwann cells. To prove this hypothesis, we screened the *Cx32* promoter for the Nbox motif (5′-CACNAG-3′), shown to be a preferential binding motif for Hes proteins [35], and found two evolutionary conserved potential sites, Nbox1 and Nbox2. In EMSAs, Hes1-containing protein extracts showed robust binding to an Nbox2- but not to an Nbox1-containing oligonucleotide (Figure 3h). To verify the functional relevance of this binding, luciferase reporter assays were performed on reporter constructs carrying mutated versions of the Nbox1 and Nbox2 motifs or mutations of both Nbox motifs in the context of the *Cx32* promoter. Only the mutation of Nbox2, solely or in combination with the mutation of Nbox1, was able to attenuate the Hes1 repressive effect on Sox10 activation (Figure 3i).

**Figure 4:**
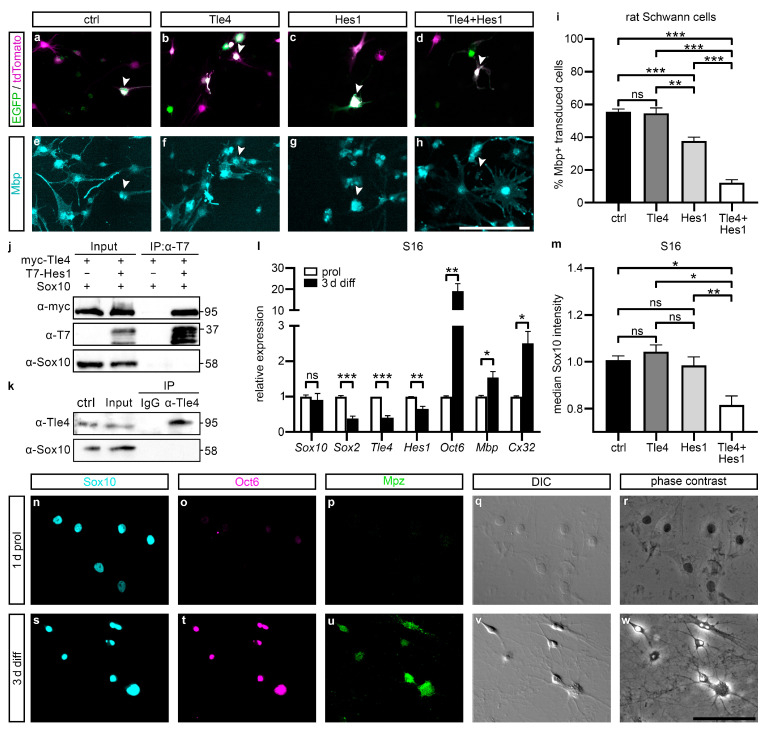
Forced expression of Tle4 and Hes1 in primary rat Schwann cells represses their differentiation. (**a**–**i**) Primary rat Schwann cells were transduced with an EGFP and tdTomato only virus (ctrl, black bar), a Tle4-EGFP and tdTomato only virus (Tle4, dark grey bar), a Hes1-tdTomato and EGFP only virus (Hes1, light grey bar) or a Tle4-EGFP and Hes1-tdTomato virus (Tle4 + Hes1, white bar). (**a**–**h**) Representative images of transduced primary rat Schwann cells differentiated for 4 days and stained for Mbp (cyan). Fluorescence of retrovirally expressed EGFP and tdTomato is shown in green and magenta. Scale bar: 100 µm. Arrowheads mark double-transduced cells. (**i**) Percentage of Mbp-positive cells of all double-transduced Schwann cells. Data represent mean values ± SEM. Statistical analysis was performed with a one-way ANOVA with Tukey’s multiple comparisons tests (ns *p* > 0.05, ** *p* ≤ 0.01, *** *p* ≤ 0.001). (**j**,**k**) Co-immunoprecipitations were performed with lysates of HEK293T cells overexpressing myc-Tle4 and Sox10 with or without additional T7-Hes1 (**j**), or with lysates of primary rat Schwann cell cultures in proliferative conditions (**k**). Precipitations were performed with anti-T7 antibody (**j**) or anti-Tle4 antibody (**k**). Subsequently, immunoblotting was performed to detect Hes1 (anti-T7 antibody), Tle4 (anti-myc antibody) or Sox10 (anti-Sox10 antiserum). The control lane (ctrl) contains lysates of HEK293T cells overexpressing myc-Tle4 and Sox10 for easy identification of endogenous protein bands (**k**). Numbers on the right indicate the detected molecular weight in kDa. (**l**) S16 cells were cultured in proliferative (white bars) and in differentiating conditions for 3 days (black bars), comparable to primary Schwann cells. The transcript levels of Schwann cell lineage markers specific to the immature state and the differentiated state were quantified in whole RNA isolations using qRT PCR. Data represent mean values ± SEM. Statistical analysis was performed with Student’s two-tailed *t*-tests (ns *p* > 0.05, * *p* ≤ 0.05, ** *p* ≤ 0.01, *** *p* ≤ 0.001). (**m**) S16 cells were transfected with a pCMV5 empty vector (ctrl, black bar), a pCAG-myc-Tle4-IRES-EGFP vector (Tle4, dark grey bar), a pCMV5-T7-Hes1 vector (Hes1, light grey bar), or both expression vectors (Tle4 + Hes1, white bar) and differentiated for 3 days. Immunocytochemistry was performed for Sox10, T7-Hes1, and EGFP as a proxy for Tle4, and Sox10 intensity was measured by flow cytometry in EGFP and T7 double positive cells. Data represent median values ± SEM. Statistical analysis was performed with a one-way ANOVA with Tukey’s multiple comparisons tests (ns *p* > 0.05, * *p* ≤ 0.05, ** *p* ≤ 0.01). (**n**–**w**) Representative immunocytochemical stainings, differential interference contrast (DIC), and phase contrast images of S16 cells kept for 1 day in proliferative conditions (1 d prol) or for 3 days in differentiating conditions (3 d diff) using antisera directed against Sox10 (cyan), Oct6 (magenta), and Mpz (green). Scale bar: 100 µm.

To provide evidence that this repression of Sox10 targets is functional during Schwann cell differentiation and necessary to prevent premature myelin gene expression, we employed primary cultures of rat Schwann cells [36], which were infected with retroviral vectors encoding Tle4-IRES-GFP and Hes1-IRES-tdTomato, alone or in combination, or with the respective GFP and tdTomato only control viruses. After 4 days of in vitro differentiation (4 d diff), the percentage of Mbp-positive cells within the tdTomato/GFP-double-positive population was analyzed as a readout of terminal differentiation and myelin gene expression in transduced cells. Whereas 56% ± 2% and 55% ± 3% of all Schwann cells were positive for Mbp in the control population and the “Tle4 only” population, Hes1 alone reduced the Mbp-positive population to 38% ± 2%. Intriguingly, a combination of both Tle4 and Hes1 lowered the fraction of Mbp-positive cells even further to 12% ± 2% (Figure 4a–i). Therefore, a synergistic repressive effect of both proteins is also detectable on endogenous myelin gene expression in primary Schwann cells.

### 2.4. Tle4 and Hes1 Inhibit Sox10 Expression and Activity in Undifferentiated Schwann Cells

To identify the mechanisms how Hes1 and Tle4 prevent precocious Sox10-dependent activation of Schwann cell maturation markers, we analyzed the protein interactions of Sox10 with Hes1 and Tle4 in co-immunoprecipitations using overexpression extracts from transfected HEK293T cells and from primary rat Schwann cells endogenously expressing Tle4 and Sox10 in proliferating conditions. Since protein–protein interactions were shown for the closely related Sox9 and Tle4 in the context of melanocyte development, the interaction of Sox10 and Tle4 appeared likely. However, neither Tle4 nor Hes1 were able to interact with Sox10 in overexpression extracts nor Tle4 and Sox10 in extracts prepared from primary rat Schwann cells in our standard setup or under widely varying experimental conditions, such as different salt and detergent concentrations as well as different precipitating antibodies. The results from co-immunoprecipitations using our established standard setup are shown as an example (Figure 4j,k). Therefore, the most parsimonious explanation is that binding of Hes1 and Tle4 to regulatory regions in the vicinity of Sox10 is sufficient to repress Sox10 activity in the absence of a direct interaction of the repressors with Sox10.

A second independent mechanism, how Tle4 and Hes1 could interfere with the Sox10 activation of Schwann cell maturation factors, is by reduction of the Sox10 protein expression in undifferentiated Schwann cells. To elucidate if Sox10 expression is indeed regulated by Tle4 and Hes1, we used S16 Schwannoma cells and quantified the signal intensity of endogenous Sox10 immunofluorescence in these cells by flow cytometry after transfecting them with control plasmids or plasmids encoding Hes1, Tle4, or both. We decided to use S16 cells for our experiments since they were transfected with substantially higher efficiency than primary Schwann cells. When cultured in the same medium as primary Schwann cells, the exchange of growth factors by dibutyryl-cAMP and high insulin concentrations triggered comparable differentiation-associated changes of cell morphology and gene expression profile in the S16 cells as in the primary Schwann cells (Figure 4l,n–w). On the transcript level, maturation genes such as *Oct6*, *Mbp*, and *Cx32* were significantly induced after 3 days of in vitro differentiation, whereas immaturity factors, such as Sox2, Tle4, and Hes1, were downregulated (Figure 4l). In immunocytochemical stainings, Sox10-, Oct6-, and Mpz-specific signal intensity was increased concomitant with a more elaborate cell morphology (Figure 4n–w). When S16 cells were transfected with Tle4 or Hes1, Sox10 signal intensity remained unaffected after 3 days of differentiation (Figure 4m). However, upon combined expression of both repressor proteins, we observed a drastic reduction of Sox10 signal intensity (Figure 4m). This hints towards a negative regulation of *Sox10* expression by Tle4 and Hes1.

**Figure 5:**
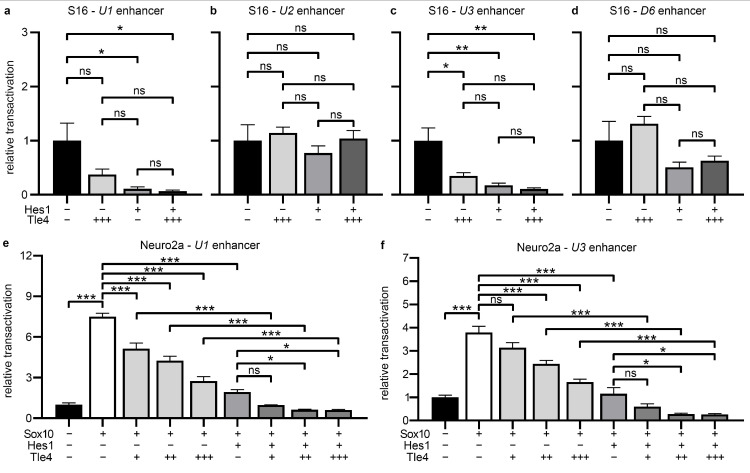
Tle4 and Hes1 repress activity of *Sox10* enhancers active during Schwann cell development. (**a**–**f**) Luciferase assays were performed in S16 cells (**a**–**d**) or Neuro2a cells (**e**,**f**) transiently transfected with luciferase reporter constructs containing the *Sox10* enhancers *U1*, *U2*, *U3*, or *D6* and a β-globin minimal promoter (TATA) upstream of the firefly luciferase open reading frame. Co-transfections with empty pCMV5 expression vectors (−), varying amounts of expression vectors (+, ++, +++) for Sox10, Tle4, and Hes1 or a combination of them were performed as indicated. Reporter gene expression was measured and the mean relative transactivation rates ± SEM are depicted. Transfections with the pCMV5 vector only were arbitrarily set to 1. Statistical analysis was performed with a one-way ANOVA with Tukey’s multiple comparisons tests between meaningful groups (ns *p* > 0.05, * *p* ≤ 0.05, ** *p* ≤ 0.01, *** *p* ≤ 0.001).

Sox10 expression in different tissues and developmental stages is regulated by various evolutionary conserved *Sox10* enhancers located upstream and downstream of the *Sox10* transcriptional start site [37,38,39]. Three *Sox10* upstream (U) enhancers and one downstream (D) enhancer with particularly high activity in Schwann cells are *U1*, *U2*, *U3*, and *D6* [39]. Those enhancers in combination drive robust Sox10 expression in Schwann cells and, at least for the *U3* enhancer, a positive feedback regulation by Sox10 was found in vitro and in vivo leading to a sustained *Sox10* transcript expression in already Sox10-positive cells [38]. Using reporter constructs in which luciferase expression is under the control of a *ß-globin* minimal promoter and *U1*, *U2*, *U3*, or *D6*, we further analyzed the effect of Tle4 and Hes1 on these enhancers and the regulation of their activity. We found strong repression of *U1* enhancer activity by Hes1 and of the *U3* activity by Tle4 or Hes1 in Sox10-positive S16 Schwannoma cells (Figure 5a,c). A tendency for additive repressive effects of both factors in combined transfections was clearly visible as well (Figure 5a,c). Comparable repressive effects were not visible for Tle4 on the *U2* and the *D6* enhancer constructs, and only a tendency for weakened repression was detectable for Hes1 alone on the *D6* enhancer (Figure 5b,d). In order to analyze transcriptional repression of the *Sox10* enhancers in more detail, further luciferase reporter assays were performed on the *U1* and *U3* containing constructs in Neuro2a cells, where enhancer activity had to be induced by Sox10 transfection. Not only the *U3* but also the *U1* enhancer was robustly activated by Sox10 in Neuro2a cells, indicating that positive autoregulation of Sox10 expression may not only take place via the *U3* [38] but also via the *U1* enhancer in the Schwann cell lineage (Figure 5e,f). Intriguingly, the Sox10 transactivation capacity on both enhancers was repressed by Tle4 and by Hes1 in this experimental setup (Figure 5e,f). An additive repressive effect was furthermore detectable in the presence of both factors.

## 3. Discussion

Sox10 was predominantly described as a regulator of Schwann cell survival and Schwann cell differentiation. In this context, it was shown to induce transcriptional regulators of Schwann cell differentiation, such as Oct6 and Egr2, and synergistically with them, activate Schwann cell maturation genes and myelin genes during developmental PNS myelination and remyelination [2,3,4,5]. Here, we focused on the novel role of Sox10 as an activator of differentiation inhibitors during the early phases of Schwann cell development. We screened for potential Sox10 targets that showed binding by Sox10 in the vicinity of their genomic locus in published ChIP-sequencing data sets on peripheral nerve tissue and strong downregulation upon terminal differentiation of Schwann cells in published RNA-sequencing data sets [17,18]. With Tle4, we identified a transcriptional regulator as a novel Sox10 target. We were able to show that Sox10 induces Tle4 expression by direct binding to a novel evolutionary conserved enhancer and that mutation of one conserved Sox binding site in this enhancer strongly reduced Sox10 transactivation capacity. Tle4, in concert with the transcriptional repressor Hes1, was in turn able to synergistically reduce Sox10 activation of known differentiation-related Sox10 targets in two ways. In one mode of action, both regulators strongly repress Sox10 transcriptional activation of regulatory regions of maturation factors and myelin genes, such as *Egr2*, *Cx32*, and *Pmp22*. This negative feedback loop of Tle4 and Sox10 resembles the regulatory interactions of Sox10 and inhibitors of differentiation in myelinating oligodendrocytes, the CNS counterpart of myelinating Schwann cells in the PNS [40,41]. In oligodendrocyte progenitor cells, Sox10 is able to directly activate the expression of Id proteins, Wnt7a and Tgfβ, which in turn inhibit oligodendrocyte differentiation. To overcome this blockade in lineage progression, Sox10-dependent activation of those repressors has to be repressed by further transcriptional repressors including Sip1 or Zfp276 [40,42]. Also in the PNS, Sip1 was shown to act on repressors of Schwann cell differentiation, and it therefore represents a good candidate to implement the derepression of Tle4/Hes1-mediated inhibition of Schwann cell differentiation [43].

Mechanistically, our data suggest that interference with Sox10 transactivation requires the binding of the Hes1/Tle4 complex in close vicinity to Sox10 binding sites in the Sox10-dependent gene regulatory regions. This hypothesis was supported by a loss of repressive activity in reporter gene assays using a *Cx32* promoter construct, where the Hes1 binding Nbox2 was mutated to a non-consensus sequence.

Interestingly, direct physical interaction of either of the two repressors with Sox10 could not be detected in co-immunoprecipitations using overexpression extracts or primary Schwann cell extracts with endogenous expression of all analyzed factors. This is in contrast to the mechanism described for the Hes1-related corepressor Hes5 in myelinating oligodendrocytes of the CNS [44]. Liu and colleagues could show that Hes5 also reduces the differentiation potential of Sox10 during CNS developmental myelination by interfering with the Sox10 activation of myelin gene expression. In oligodendrocyte progenitor cells, Hes5 is activated by active Notch signaling and interferes not only with Sox10 transcription but also prevents it from binding to its downstream target genes by sequestration. Overexpression of Hes5 in oligodendroglial cells therefore reduced bioactive levels of Sox10 and the expression of myelin genes in an indirect as well as in a direct mode of action. As an example for the direct mode, Hes5 binding to the *Mbp* promoter led to the local recruitment of repressive chromatin remodeling complexes. Interestingly, in the same experimental setup, Hes1 was not able to reduce myelin gene expression in oligodendroglial cells, though it was expressed endogenously [37]. This points to differential functions of both Hes proteins in myelinating glia of the CNS and PNS, despite the fact that both proteins target Sox10 as a central hub of myelin gene transcriptional regulation. Obviously, it is possible that only Hes5, but not Hes1, directly interacts with Sox10, and that this difference is the reason for the missing repressive activity of Hes1 in oligodendrocytes. In contrast, the knockdown of Hes5 in primary Schwann cell cultures was able to increase their differentiation capacity [45], despite the fact that endogenous expression of *Hes5* transcripts was almost undetectable in RNA-sequencing data on primary Schwann cell cultures from another study [17]. Due to those partially conflicting in vitro findings, conditional knockout mouse mutants would have to be generated and analyzed to fully unravel the in vivo roles of both Hes proteins and their differential interactions with Tle4 and Sox10.

Additionally, we could not detect a sumoylation-dependent repressive activity of Sox10 or an interaction of Sox10 with Tle4, as previously described for the closely related Sox9 protein in the context of melanocyte development [25]. Tle4 interfered with the Sox10-dependent activation of Schwann cell maturation factors, even when a non-sumoylatable Sox10 was used. All these results point to a mode of action were Tle4 and Hes1 do not directly interact with Sox10 or directly impede its binding to target gene regulatory regions. Tle proteins were shown to shut down gene expression of their target genes using different modes of action (for detailed information, see [46]). Depending on their binding partners they may be recruited to their target genes by DNA-binding transcriptional repressors, such as Hes proteins, and together with them, recruit HDACs or other silencing complexes generating a non-accessible chromatin landscape for activating transcription factors. Another mechanism is the specific binding of Tle proteins to Tcf/Lef proteins as Wnt effectors that results in the displacement of ß-Catenin from the Tcf/ß-Catenin complex and prevents Wnt target activation [47]. A Sox10-independent mechanism of the Tle4-mediated repression of maturation factors in vivo may also involve the repression of Wnt target genes, since Wnt signaling was implicated in axonal sorting and myelin gene expression during Schwann cell maturation and PNS myelination [48,49].

Hes1 was also described to interfere with transcriptional elongation in the context of immune gene activation by blocking the recruitment of the p-TEFb complex [50]. Interestingly, Sox10 was shown to directly interact with this positive transcriptional elongation factor in Schwann cells and to recruit it to target genes to enable the productive transcription of maturation and myelin genes. Blocking of transcriptional elongation in this context led to the reduced expression of the aforementioned maturation factors [51]. If Hes1 and Tle4 are also able to interfere with the p-TEFb-mediated positive regulation of transcriptional elongation on Schwann cell maturation genes, direct binding to Sox10 would not be necessary. Instead, this mode of action would postulate a context-dependent gene-intrinsic recruitment of Hes1 and Tle4 to Sox10-activated genes and the repression of productive gene transcription.

A novel mode of action that we identified for Tle4 and Hes1 in order to suppress premature Schwann cell differentiation was the direct repression of Sox10 expression by interference with activation of the *Sox10* enhancers *U1* and *U3*. At least partially, this may be causative for the reduced Sox10 protein levels measured in Hes1/Tle4-transfected S16 Schwannoma cells. A similar effect was previously shown for the Hes1-related protein Hes5 in oligodendrocyte precursor cells during CNS development [44]. Nonetheless, regulation of Sox10 translation or protein stability might also take place in a parallel mode of action, similar to different proteins of the Fanc protein family that are stabilized by Hes1 in a Notch-dependent way [52].

Interestingly, not all *Sox10* enhancers that are active during Schwann cell development were changed in their activity by Tle4 and Hes1 in our reporter gene assays. Among the unresponsive enhancers, *D6* is only active in late Schwann cell development and *U2* is also important to drive oligodendroglial Sox10 expression in the CNS [53]. Failure to suppress *U2* and *D6* enhancer activity may thus hint towards a specific regulation by Tle4 and Hes1 of *Sox10* enhancers that are already active in early Schwann cell development and selectively active in peripheral glia. Autoregulation by Sox10 appears to be another common denominator for enhancers that are responsive to Tle4 and Hes1. In this respect, it is worth mentioning that *U2* was only weakly bound by Sox10 in gel shift assays, arguing against an autoregulatory function of this specific enhancer [39].

Taken together, we unraveled specific functions of Sox10 and its newly identified regulatory target Tle4 during the early stages of Schwann cell development. We demonstrated a novel regulatory feedback mechanism by which Tle4 and Hes1 prevent premature Schwann cell differentiation in two ways, i.e., by antagonizing Sox10 activity and repressing Sox10 expression in the early stages of Schwann cell development. Repression of pro-differentiative cues also is essential after peripheral nerve injury, when differentiated Schwann cells transdifferentiate in so-called repair Schwann cells. Many inhibitors of developmental Schwann cell differentiation are transiently upregulated again, and inhibitory pathways are transiently reactivated, including the Hes1-inducing Notch pathway that has to be shut down again to allow efficient remyelination [54,55]. Whether the feedback loop described for Tle4, Hes1, and Sox10 is also active during de- and remyelination events in the PNS will have to be disclosed in future experiments.

## 4. Materials and Methods

### 4.1. Transgenic Animal Studies, Immunohistochemistry, and Immunocytochemistry

All animal studies were conducted according to animal welfare laws and both male and female mice were used for analysis indiscriminately. Mice were on a C3H background and kept under a 12-h light–dark cycle. Mice were bred overnight and the day a vaginal plug was detected, defined as 0.5 days *post coitum* (dpc). Embryos were obtained at 13.5 dpc, 16.5 dpc, and 18.5 dpc and fixed in 4% PFA for 4 h to overnight at 4 °C. Tissue was dehydrated in 30% sucrose in PBS, mounted in TissueTek Freezing Medium (Leica Wetzlar, Germany) and sectioned transversally at 10 µm. For immunohistochemical stainings, the following antibodies were used: guinea pig anti-Sox10 antiserum (dilution 1:500, self-made, RRID: AB_2721917, [56]), rabbit anti-Sox2 (dilution 1:500, self-made, [57]), goat anti-Sox2 (dilution 1:500, Santa Cruz sc-17320, Dallas, TX, USA), rabbit anti-Oct6 (dilution 1:2000, self-made, RRID:AB_2891333, [58]), rabbit anti-Egr2 (dilution 1:200, Covance #PRB-236P, Princeton, NJ, USA), and mouse anti-Tle4 (dilution 1:200, Santa Cruz sc-365406, Dallas, TX, USA). The mouse anti-Tle4 antibody signal was amplified using the TSA Plus Fluorescein System (PerkinElmer, Waltham, MA, USA) according to the manufacturer’s instructions after citrate buffer-mediated antigen retrieval.

For immunocytochemistry, cells were fixed in 3% PFA for 15 min at room temperature. The following antibodies were used: goat anti-Sox10 (1:1000, self-made, RRID: AB_2891326, [36]), goat anti-tdTomato (dilution 1:500, SICGEN AB8181-200, Cantanhede, Portugal), chicken anti-GFP (dilution 1:2000, Aves GFP-1020, Davis, CA, USA), rat anti-Mbp (dilution 1:750, Serotec MCA409S, Neuried, Germany), rabbit anti-Oct6 (dilution 1:2000, self-made, RRID: AB_2891333, [58]), and chicken anti-Mpz (dilution 1:2000, Aves AB_2313561, Davis, CA, USA).

Secondary antibodies were coupled to Alexa488 (dilution 1:500, Molecular Probes, Eugene, OR, USA), CF488 (dilution 1:2000, Hölzel B-20166, Cologne, Germany), Cy3, Cy5, or AMCA (dilution 1:200 for Cy3 and Cy5, 1:100 for AMCA, all Dianova, Eching, Germany). Nuclei were stained with DAPI if AMCA-coupled antibodies were not used. Samples were analyzed on a Leica DMI 6000B inverted microscope equipped with a DFC 360FX camera (Leica, Wetzlar, Germany).

### 4.2. Culture of Primary Rat Schwann Cells and Cell Lines, Transfections, and Viral Transduction

Primary rat Schwann cells were prepared and maintained in the defined medium as described [36]. For differentiation of primary rat Schwann cells and S16 cells, the defined medium was supplemented additionally with 1 mM dibutyryl-cAMP and 5.7 µg/mL insulin. Whole-cell lysates of primary rat Schwann cells were prepared as described before [59].

Neuro2a neuroblastoma (CCL-131, ATCC, Manassas, VA, USA), S16 Schwannoma (CRL-2941, ATCC, Manassas, VA, USA), and HEK293T cells (CRL-1573, ATCC, Manassas, VA, USA) were maintained in high glucose DMEM (Gibco, Waltham, MA, USA) supplemented with 10% FCS (Gibco, Waltham, MA, USA) and penicillin/streptomycin (Gibco, Waltham, MA, USA).

For luciferase reporter assays in S16 and Neuro2a cells, 0.5 µg of luciferase reporter plasmid and up to 0.375 µg of pCMV5-based expression plasmids were transfected using Xfect (TaKaRa Bio, Kusatsu, Japan) according to the manufacturer’s instructions per well in 24-well plates. Empty pCMV5 was used to keep overall plasmid amounts constant. The luciferase assay has been described in detail [59]. Reporter gene activity was measured 2 days post transfection.

For protein interaction and electrophoretic mobility shift assays, HEK293T cells were transfected in 10-cm dishes with 10 µg pCMV5-based expression plasmids using polyethylenimine. Subsequently, whole cell lysates were prepared as described [59].

Retroviruses were produced by transient transfection of the HEK293-derived packaging cell line 293GPG [60] with retroviral expression plasmids using PolyJet according to the manufacturer’s protocol (SignaGen, Frederick, MD, USA). Retroviruses were enriched by ultracentrifugation and used for transduction as described before [61]. Viral titers were determined in HEK293T cells by infection with serial dilutions of viral preparations. Two days post infection, titers of infectious units/mL were determined by quantifying the number of EGFP or tdTomato-positive cells using flow cytometry (FACS Calibur, BD Biosciences, Franklin Lakes, NJ, USA). Expression of encoded transgenes was confirmed using immunocytochemical staining of transduced cells. Primary rat Schwann cells were transduced at an MOI of 5 for each individual virus, maintained in the proliferative defined medium. The next day, the medium was changed to the defined medium with high dibutyryl-cAMP concentrations and insulin to induce differentiation. Cells were kept in the differentiation medium for 4 days with a medium change on day 2 and fixed at 4 days of differentiation.

### 4.3. Plasmids

Retroviral expression plasmids pCAG-myc-IRES-EGFP [40] and pCAG-IRES-tdTomato [62] were described before. pCAG-myc-Tle4-IRES-EGFP was produced by inserting the BsaI-digested murine Tle4 open reading frame into BamHI-digested pCAG-myc-IRES-EGFP. pCAG-flag-Hes1-IRES-tdTomato was produced by cloning the murine Hes1 (NM_008235.3) coding sequence in frame with an N-terminal flag-tag into pCAG-IRES-tdTomato via BamHI and BglII.

The pCMV5-Sox10 and pCMV5-Sox10-MIC vectors were described before [22]. pCMV5-Sox10 K55/246/357R was generated by site-directed mutagenesis with the QuickChange XL Site-Directed Mutagenesis Kit (Agilent, Santa Clara, CA, USA). The murine Tle4 coding sequence (NM_011600.4) was amplified and inserted in frame with an N-terminal myc-tag into pCMV5 via SalI and KpnI. The murine Hes1 coding sequence (NM_008235.3) was inserted in frame with an N-terminal T7-tag into pCMV5 via BamHI and SalI to generate pCMV5-T7-Hes1.

pBKS- and pTATAluc-based luciferase reporter plasmids Cx32-416-luc (*Cx32* promoter), pTATAluc-Pmp22 intronic enhancer (*Pmp22* enhancer), pTATAluc-Krox20MSE, and *Sox10* enhancer reporter plasmids *U2-luc* and *U3-luc* were described before [5,24,38,39,53,63]. Comparable to the aforementioned *Sox10* enhancers *U2* and *U3*, the previously described *Sox10* enhancer regions *U1* and *D6* were cloned upstream of a *ß-globin* minimal promoter and the luciferase reporter into pTATAluc to generate *U1-luc* and *D6-luc* [39]. The analyzed *Tle4* ECRs ECR-78 kb (rn6: chr1:231,608,621-231,609,620) and ECR-19 kb (rn6: chr1:231,550,132-231,550,931) were both amplified from rat genomic DNA and inserted into pTATAluc via KpnI and XhoI. Mutagenesis of the conserved Sox10 binding site in ECR-78 kb was performed via site-directed mutagenesis with the Q5^®^ Site-Directed Mutagenesis Kit (NEB, Ipswich, MA, USA). In general, plasmid identity and correctness of insert sequences were validated by restriction digest and Sanger sequencing. Additionally, correct expression of encoded proteins was confirmed by Western blotting.

### 4.4. Flow Cytometry

For flow cytometric analysis of endogenous Sox10 levels, S16 cells were transfected with pCMV5-T7-Hes1 and pCAG-myc-Tle4-IRES-EGFP or the appropriate empty control plasmid using Xfect (TaKaRa Bio, Kusatsu, Japan), as described above. The next day, the medium was changed to the defined medium with 1 mM dibutyryl-cAMP and 5.7 µg/mL insulin and the cells were allowed to differentiate for 3 days. Subsequently, cells were detached using trypsin/EDTA, fixed with 4% PFA for 15 min in suspension, and permeabilized using PBS with 0.1% Triton X-100. Unspecific binding of antibodies was blocked with 10% FCS, 1% BSA, and mouse total IgG (dilution 1:100). Cells were stained with goat anti-Sox10 antiserum (dilution 1:200, self-made, RRID: AB_2891326, [36]), rabbit-anti GFP-AF488 antibody (dilution 1:200, A21311, Molecular Probes, Eugene, OR, USA), and mouse anti-T7 antibody (dilution 1:1000, Merck, Darmstadt, Germany). Secondary antibodies coupled to Cy3 and Cy5 were used (dilution 1:200, Dianova, Eching, Germany). The Sox10 median fluorescence intensity was subsequently analyzed on a LSRFortessa™ Cell Analyzer (BD Biosciences, Franklin Lakes, NJ, USA) in only the transfected cell population (indicated by GFP and T7-Cy3 fluorescence, respectively). Data were analyzed with Flowing Software 2 (Turku Bioscience, Turku, Finland).

### 4.5. Immunoprecipitation Studies and Western Blotting

Whole cell lysates of HEK293T cells and primary rat Schwann cells in proliferative conditions were used for co-immunoprecipitation studies. Whole cell lysates of primary rat Schwann cells in proliferative conditions or after 2 days of in vitro differentiation were used for expression studies. For co-immunoprecipitation studies, proteins of interest were precipitated with mouse a-myc antibody (clone 9B11, Cell Signaling, Danvers, MA, USA), mouse anti-T7 antibody (AB3790, Merck, Darmstadt, Germany) or mouse anti-Tle4 antibody (sc-365406, Santa Cruz, Dallas, TX, USA), and Protein A- or Protein G-sepharose beads (#17-0780-01, Lot #10254134 and #17-0618-01, Lot #10244249, GE Healthcare, Chicago, IL, USA). Proteins were eluted from beads by boiling in Laemmli buffer (50 mM Tris-HCl, 2% SDS, 1% β-Mercaptoethanol, 10% Glycerol, 0.1% Bromophenol blue) at 95 °C for 5 min. Samples from immunoprecipitation studies and whole cell lysates were separated on 10% polyacrylamide gels, and after semi-dry Western blotting on nitrocellulose membranes, proteins were detected with the following antibodies: rabbit anti-Sox10 (dilution 1:4000, self-made [64]), mouse anti-myc (dilution 1:4000, Cell Signaling 9B11, Danvers, MA, USA), mouse anti-T7 (dilution 1:4000, Merck AB3790, Darmstadt, Germany), mouse anti-Tle4 (dilution 1:200, Santa Cruz sc-365406, Dallas, TX, USA), guinea pig anti-Egr2 (dilution 1:20000, self-made RRID:AB_2891327, [58]), rabbit anti-Gapdh (dilution 1:200000,#10494-1-AP, Lot # 00103483, Proteintech, IL, USA), and rabbit anti-Gapdh (dilution 1:4000, Santa Cruz sc-25778, Dallas, TX, USA). Blots were developed with goat anti-mouse-HRP (#074-1506, Lot #130535, 1:2000 dilution, KPL, Gaithersburg, MD, USA) and Protein A-HRP conjugates (1:2000 dilution, Zymed, Vienna, Austria) using enhanced chemiluminescence via Luminol (A4685, Sigma-Aldrich, St. Louis, MO, USA).

### 4.6. Electrophoretic Mobility Shift Assays

Oligonucleotides were labelled with ^32^P as described before [64]. As an unspecific competitor, 0.05 µg salmon sperm DNA sheared to 100 to 400 bp fragments was used. Whole cell lysates from HEK293T cells transfected with a control plasmid (pCMV5) or pCMV5 expression plasmids for T7-Hes1, Sox10 or a Sox10 fragment encompassing the N-terminal DNA binding domain of rat Sox10 (Sox10aa1-189 = Sox10-MIC [22]) were co-incubated with labelled oligonucleotides.

### 4.7. qRT PCR Analysis

Total RNA was harvested from cultured primary rat Schwann cells and S16 cells using Trizol (15596026, Invitrogen, Waltham, MA, USA) or using the Qiagen RNeasy Micro kit (74004, Qiagen, Venlo, Netherlands). Reverse transcription was performed with an oligo-dT primer and an M-MuLV reverse transcriptase (NEB, Ipswich, MA, USA). All RNA samples were tested negative for DNA contaminations using a negative control sample without M-MuLV reverse transcriptase in reverse transcription reactions and primers, which do not span exon–exon borders. In those samples, we did not detect any amplification of the test amplicon when performing quantitative PCR for 45 cycles. Quantitative PCR was performed using PowerUp SYBR Green Mastermix (A25743, Thermo Fisher Scientific, Waltham, MA, USA) with the following oligonucleotides and amplicon sizes: CACTGTCCATCTGGGACCTG and CCAAACCACCTGTCCAGAGC for *Tle4*, 252 bp, spans exon–exon junction; GTGAAGCACCTCCGGAACCT and GGTAGGTCATGGCGTTGATC for *Hes1*, 214 bp, spans exon–exon junction; AGCGCATCCAGTGGGTAGGG and CCCGAGGATGCCACCGATCA for *Mpz*, 212 bp, spans exon–exon junction; GCAAGGACAGCGAAAAAGAC and GCTGAGATGGCTCGAGAAAC for *Egr2*, 175 bp, does not span exon–exon junction; GTCAGATGGGAACCCAGAGCAC and CCCGTAGCCAGCTGCCGAG for *Sox10*, 289 bp, spans exon–exon junction; CACAACTCGGAGATCAGCAA and CTCCGGGAAGCGTGTACTTA for *Sox2*, 190 bp, spans exon–exon junction; CTGCAACTGGAGAAGGAGGT and GTGGTGATGGTGGTGGTGTA for *Oct6*, 207 bp, does not span exon–exon junction; CCAAGTTCACCCCTACTCCA and TAAGTCCCCGTTTCCTGTTG for *Mbp*, 480 bp, spans exon–exon junction; TCCATCAAACCTTCCCTCCC and TCAGGGAGAGGGAAGAGGAA for *Cx32*, 164 bp, does not span exon–exon junction; TCCAGTATGACTCTACCCACG and CACGACATACTCAGCACCAG for *Gapdh*, 148 bp, spans exon–exon junction; GTTCGTGTACTGCGGCAAGA and ACAGGATTCATGGCCACACC for *Rpl8*, 363 bp, spans exon–exon junction. All samples were processed as technical triplicates. Data was analyzed by the ΔΔCt method by normalizing to Rpl8 and Gapdh.

### 4.8. Quantifications and Statistical Analysis

Independent experiments, separately generated samples or results from different animals were treated as biological replicates. Sample size was *n* ≥ 3 for all experiments. GraphPad Prism7 (GraphPad software, La Jolla, CA, USA) was used for statistical testing using two-tailed Student’s *t* tests and analyses of variance (ANOVA) with Tukey’s multiple comparisons tests to determine differences in cell numbers, luciferase activity, median fluorescent intensities, or transcript levels (ns *p* > 0.05; * *p* ≤ 0.05; ** *p* ≤ 0.01, *** *p* ≤ 0.001). The variance between statistically compared groups was similar and the data displayed a normal distribution.

## Data Availability

All data generated or analyzed during this study are included in this published article.
